# The Use of Induced Pluripotent Stem Cells to Study the Effects of Adenosine Deaminase Deficiency on Human Neutrophil Development

**DOI:** 10.3389/fimmu.2021.748519

**Published:** 2021-10-28

**Authors:** Michael Tsui, Weixian Min, Stephanie Ng, Kerry Dobbs, Luigi D. Notarangelo, Yigal Dror, Eyal Grunebaum

**Affiliations:** ^1^ Developmental and Stem Cell Biology Program, Hospital for Sick Children, Toronto, ON, Canada; ^2^ The Institute of Medical Sciences, The University of Toronto, Toronto, ON, Canada; ^3^ Genetics and Genome Biology Program, The Hospital for Sick Children, Toronto, ON, Canada; ^4^ Laboratory of Clinical Immunology and Microbiology, Division of Intramural Research, National Institute of Allergy and Infectious Diseases, National Institutes of Health, Bethesda, MD, United States; ^5^ Marrow Failure and Myelodysplasia Program, Division of Hematology/Oncology, Department of Pediatrics, The Hospital for Sick Children, Toronto, ON, Canada; ^6^ Division of Immunology and Allergy, Department of Pediatrics, The Hospital for Sick Children, Toronto, ON, Canada

**Keywords:** neutropenia, ribonucleotide reductase (RNR) inhibitors, iPSC (induced pluripotent stem cell), adenosine deaminase (ADA) deficiency, multipotent

## Abstract

Inherited defects that abrogate the function of the adenosine deaminase (ADA) enzyme and consequently lead to the accumulation of toxic purine metabolites cause profound lymphopenia and severe combined immune deficiency. Additionally, neutropenia and impaired neutrophil function have been reported among ADA-deficient patients. However, due to the rarity of the disorder, the neutrophil developmental abnormalities and the mechanisms contributing to them have not been characterized. Induced pluripotent stem cells (iPSC) generated from two unrelated ADA-deficient patients and from healthy controls were differentiated through embryoid bodies into neutrophils. ADA deficiency led to a significant reduction in the number of all early multipotent hematopoietic progenitors. At later stages of differentiation, ADA deficiency impeded the formation of granulocyte colonies in methylcellulose cultures, leading to a significant decrease in the number of neutrophils generated from ADA-deficient iPSCs. The viability and apoptosis of ADA-deficient neutrophils isolated from methylcellulose cultures were unaffected, suggesting that the abnormal purine homeostasis in this condition interferes with differentiation or proliferation. Additionally, there was a significant increase in the percentage of hyperlobular ADA-deficient neutrophils, and these neutrophils demonstrated significantly reduced ability to phagocytize fluorescent microspheres. Supplementing iPSCs and methylcellulose cultures with exogenous ADA, which can correct adenosine metabolism, reversed all abnormalities, cementing the critical role of ADA in neutrophil development. Moreover, chemical inhibition of the ribonucleotide reductase (RNR) enzyme, using hydroxyurea or a combination of nicotinamide and trichostatin A in iPSCs from healthy controls, led to abnormal neutrophil differentiation similar to that observed in ADA deficiency, implicating RNR inhibition as a potential mechanism for the neutrophil abnormalities. In conclusion, the findings presented here demonstrate the important role of ADA in the development and function of neutrophils while clarifying the mechanisms responsible for the neutrophil abnormalities in ADA-deficient patients.

## Introduction

The ubiquitously expressed adenosine deaminase (ADA) enzyme irreversibly deaminates adenosine (Ado) and deoxyadenosine (dAdo) into inosine and deoxyinosine, respectively. Although ADA can also be expressed as an ectoenzyme on the surface of various cells, it is predominantly a cytoplasmic enzyme, with its highest activity detected in rapidly proliferating cells such as in the lymphoid tissue (1). Autosomal recessive inherited defects that profoundly compromise ADA enzyme activity cause severe combined immunodeficiency (ADA-SCID) with increased susceptibility to infections, autoimmunity, and malignancy (2). ADA deficiency has been shown to cause abnormal maturation and accelerated apoptosis of thymocytes, defective signaling in peripheral T cells, and abnormal regulatory T cells (3–6). It is hypothesized that ADA deficiency causes accumulation of the enzyme’s substrate, dAdo, which is phosphorylated to deoxyadenosine triphosphate (dATP) that is toxic to cells. Additionally, elevated concentration of Ado, an important signaling molecule that mediated its effects in part through the four G-protein coupled Ado receptors (AdoR), A1, A2A, A2B, and A3, has been suggested to disrupt lymphoid function (7). Depletion of ADA products could also contribute to T lineage abnormalities seen in ADA-SCID (8).

ADA-SCID patients have been shown to suffer from non-lymphoid abnormalities, including frequent lung disease (9), bone and cartilage dysplasia (10), neurologic abnormalities (11), and hearing defects (12, 13). Additionally, accumulating data suggest that neutrophils might also be affected by ADA deficiency. Recently, intermittent or persistent low neutrophil counts in peripheral blood were identified in 13 of 20 ADA-deficient infants, with the most severe neutropenia occurring during infections (14). Moreover, neutrophil abnormalities with marked neutropenia, hyperlobular and pyknotic neutrophils have been previously reported among ADA-deficient patients who received allogeneic or autologous gene-corrected hematopoietic stem cell (HSC) (15). These patients displayed dyspoietic peripheral blood neutrophils and bone marrow hypocellularity and dysplasia. Others have shown that peripheral blood CD34^+^ cells from ADA-deficient patients have decreased chemotactic potential in response to G-CSF (16). Taken together, these data suggest that ADA deficiency may impact myeloid cells, particularly when accelerated production of neutrophils is required. Nevertheless, due to limited availability of bone marrow and peripheral blood samples from ADA-deficient infants, who are often treated soon after diagnosis with enzyme replacement, such as ADA conjugated to polyethylene glycol (PEG-ADA), HSC transplantation, or gene therapy (17), it has been difficult to study neutrophils in this condition. Moreover, the possible contribution of abnormalities in the bone marrow microenvironment to the alterations observed in ADA-deficient patients (18), in addition to an intrinsic defect in hematopoietic stem cells, could not have been studied.

Allosteric inhibition of ribonucleotide reductase (RNR) enzyme by increased intracellular concentrations of dATP has previously been suggested as a potential cause for the T cell abnormalities in ADA deficiency (19). RNR is critical for the *de novo* synthesis of deoxyribonucleotides, the precursors needed for DNA synthesis replication and repair, and perturbation of RNR function can severely deplete the deoxynucleotide pool in cells (20). RNR is composed of two homodimer subunits, R1 and R2. R2 is essential for the initiation of nucleotide reduction and has been a target for many hematological malignancies treatments such as hydroxyurea (HU) (21) as well as nicotinamide (NAM) and trichostatin A (TSA) that disrupt RNR homodimer assembly (22). While the inhibition of RNR has been shown to interfere with T cell expansion, the role of RNR in neutrophil biology has not been extensively studied. Yet, 61–81% of patients with cancer treated with potent RNR inhibitors experienced neutropenia (23–28). In addition to neutropenia, treatment with HU was associated with neutrophil hypersegmentation (29) and significantly greater DNA damage (30), similar to the changes that have been observed in neutrophils from ADA-deficient patients, suggesting that disruption of RNR function might contribute to the neutrophil abnormalities seen in ADA deficiency.

Diverse somatic cells, including hematopoietic- and skin-derived cells, can be reprogrammed to generate induced pluripotent stem cells (iPSC) that are characterized by their ability to produce differentiated progeny from each of the three embryonic germ layers. Importantly, iPSC can be utilized as a renewable source of cells or differentiated toward a specific lineage of interest with the aid of defined growth conditions. Moreover, reliance on iPSC avoids the ethical issues associated with the embryonic stem cells, while providing cells from patients with well-defined phenotypes (31).

Differentiation of iPSCs *ex vivo* into multipotent hematopoietic progenitors (MHP) and subsequently into myeloid lineage cells has previously allowed a better understanding of the early and late hematopoietic defects. This strategy has been particularly beneficial in situations where peripheral blood neutrophils cannot easily be obtained, such as pediatric patients affected by reticular dysgenesis (32) or severe congenital neutropenia (33). Here, a similar strategy was employed to demonstrate for the first time that ADA deficiency directly interferes with the formation of MHPs and neutrophils from iPSCs.

## Methods

### Induced Pluripotent Stem Cells

ADA-iPSC-1 was established from skin fibroblasts of a 3-month-old male with compound heterozygous mutations in the ADA gene, c.646G>A (p.216G>R) and c.1050_1054del, previously described (34). ADA-iPSC-2 was independently created from fibroblasts of another unrelated 1-month-old male patient with identical ADA gene mutations (35). These mutations were previously shown to cause near-absent ADA activity (36). ADA-iPSC-1 generated using retroviruses expressing OCT4, SOX2, KLF4, and MYC, with pluripotency determined by expression of pluripotency markers and teratoma formation as previously described (37). ADA-iPSC-2 was generated at the Centre for Commercialization of Regenerative Medicine, Toronto, Canada, using non-integrative Sendai virus. Pluripotency was confirmed by demonstrating >97% expression of pluripotency markers OCT4, SOX2, TRA160, and SSEA4. ADA deficiency in both IPSCs were repeatedly confirmed as <1% ADA activity [measured by conversion of [^14^C]Ado, as previously described (38)] in comparison to activity of iPSCs generated from two unrelated ADA-proficient healthy controls (CTL-iPSC-1 and CTL-iPSC-2).

Early hematopoietic differentiation: Differentiation of iPSCs to early MPH was performed as previously described (32). Briefly, iPSCs were detached and suspended in base media comprised of 80% KnockOut DMEM (Thermo Fisher Scientific, 10829018, Waltham, MA, USA), 20% heat inactivated FBS (Wisent Bioproducts, 080-450, Montreal, QC, Canada), 1% NEAA (Thermo Fisher Scientific, 11140050, Waltham, MA, USA), L-glutamine (Thermo Fisher Scientific, 35050061, Waltham, MA, USA), transferrin (Sigma-Aldrich, 10652202001, Darmstadt, Germany), 2-mercaptoethanol (Sigma-Aldrich, M6250, Darmstadt, Germany), and L-ascorbic acid (Sigma-Aldrich, A92902, Darmstadt, Germany). Cells (6×10^6^ iPSCs) were seeded in ultra-low-attachment plates (Corning, 3471, Corning, NY, USA). Media was replaced after 1 day and every 3 days afterwards, using differentiation media, comprised of base media supplemented with recombinant human SCF (300-07), Flt3 (300-19), IL-3 (200-03), IL-6 (200-06), G-CSF (300-23), and BMP4 (120-05ET), all from PeproTech, Rocky Hill, NJ, USA. Embryoid bodies (EB) were collected with the media after 5, 8, 11, and 14 days, carefully dissociated into single cells using 0.25% trypsin (Wisent Bioproducts, 325-040-EL, Montreal, QC, Canada), enumerated and analyzed by flow cytometry for the expression of CD34^+^ and CD45^+^ (60013AZ and 60018PB, respectively, STEMCELL Technologies, Vancouver, BC, Canada) that characterize MHPs (39). Flow cytometry analysis was performed using the BD LSR II CFI, and data were acquired using the BD FACSDiva software and analyzed using FlowJo v10.0.7 (BD Biosciences, San Jose, CA, USA).

### Late Hematopoietic Differentiation

After 14 days, dissociated EB were resuspended at a density of 4×10^4^ cells/ml in methylcellulose (MethoCult™ H4034 Optimum, STEMCELL Technologies, Vancouver, BC, Canada) as previously described (32). After additional 12 days (day 14 + 12), hematopoietic colony-forming units (CFU) formation in the methylcellulose was assessed. Morphology of CFU was determined using an inverted light microscopy and the numbers of granulocyte CFU (CFU-G), granulocyte-monocyte CFU (CFU-GM), and granulocyte-erythroid-monocyte/macrophage CFU (CFU-GEMM), as well as erythroid CFU (CFU-E), erythroid blast-forming units (BFU-E), and monocyte CFU (CFU-M) visualized were recorded. Neutrophils were purified from CFU-G by gently dissolving the methylcellulose in PBS, followed by negative selection using the EasySep direct neutrophil isolation kit (STEMCELL Technologies, 19666, Vancouver, BC, Canada). The numbers of neutrophils were determined by hemocytometer.

### Characterization of Neutrophils

Preliminary work demonstrated that ~95% of the neutrophils, generated from iPSCs and subsequently isolated from methylcellulose, expressed CD11b, CD45, as well as CD16 and CD66b, characteristic of mature neutrophils (31), similar to neutrophils isolated from peripheral blood of healthy donors (**Supplementary Figure 1**). Neutrophils derived from iPSCs were also able to produce reactive oxidative species, determined as previously described (40) by the reduction of dihydroradamine 123 (**Supplementary Figure 2**). The morphology of the neutrophils was assessed by light microscopy following staining with May-Giemsa-Grunewald (Sigma-Aldrich Procedure No. GS-10). The percentage of hyperlobular neutrophils, defined as having six or more nuclear segmentation, among 100 randomly selected neutrophils was calculated. Viability of the neutrophils was assessed by flow cytometry using propidium iodide (PI, from Sigma-Aldrich, P4864, Darmstadt, Germany), and the percentages of PI^−^/Annexin V^+^ apoptotic cells were determined by additional annexin V staining (ThermoFisher Scientific, V13242, Waltham, MA, USA). Expression of CD11b^+^ (BD Biosciences, 557754, San Jose, CA, USA) and CD45^+^ (STEMCELL Technologies, 60018PB, Vancouver, BC, Canada) neutrophils was measured using flow cytometry. Phagocytic capability of PMA-activated neutrophils was assessed by internalization of IgG-opsonized fluorescent 1 µm microspheres (Thermo Fisher Scientific, F13083, Waltham, MA, USA) as previously described (41). The fluorescence of beads that adhered to the surface of neutrophils was quenched using 5% of 0.4% trypan blue (Thermo Fisher Scientific, 15250061, Waltham, MA, USA) immediately prior to acquisition (42), and fluorescence of internalized beads was identified using flow cytometry.

### Restoration of ADA Activity

To confirm that abnormal purine metabolism is the cause for neutrophil dysfunction and to determine whether restoring purine homeostasis will correct neutrophil development, exogenous ADA was added using PEG-ADA (pegademase bovine or the recombinant elapegademase-lvlr, Leadiant Biosciences, Pomezia, Italy). PEG-ADA (0.5 units/ml) was added to the iPSC cultures and replenished with every media change. Upon EB dissociation and resuspension in the methylcellulose, PEG-ADA concentration was increased to 1 unit/ml. One unit of activity was defined as the amount of ADA required to convert 1 µM of Ado to inosine per minute at 25°C and pH 7.3. The concentrations of PEG-ADA were chosen by titrating and evaluating their effects on differentiating iPSCs.

### RNR Inhibition

To examine the effects of RNR inhibition and identifying any similarities to the effects caused by ADA deficiency, CTL-iPSC-1 differentiation media was supplemented with 10 uM HU (Sigma-Aldrich, H8627, Darmstadt, Germany), which inhibits RNR by quenching the tyrosyl free radical, or with 20 mM NAM (Sigma-Aldrich, N0636, Darmstadt, Germany) and 5 uM TSA (Sigma-Aldrich, T8552, Darmstadt, Germany), which interfere with RNR homodimer assembly. The concentrations of RNR inhibitors were chosen as not causing increased apoptosis neutrophils as determined by Annexin V^+^/PI.

### Statistical Analysis: 

The statistical software GraphPad Prism v8.0.2 was utilized to perform statistical analysis. All data in the results and figures are represented as a mean ± standard deviation (SD). Comparative analysis was done using unpaired unequal variance Student’s t-test for two groups, and one-way ANOVA for more than two groups.

## Results

**Figure 1:**
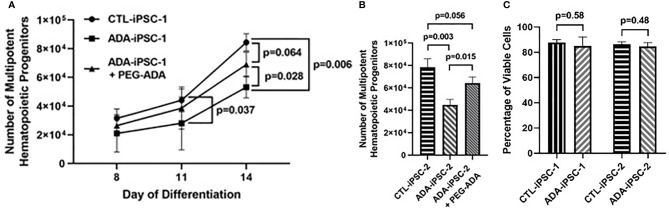
Impaired production of multipotent hematopoietic progenitors from ADA-deficient induced pluripotent stem cells. iPSCs were dissociated to form embryoid bodies (EB) and differentiated for 14 days to generate multipotent hematopoietic progenitors (MHP). **(A)** The number of CD34^+^/CD45^+^ MHPs generated from 6×10^6^ healthy control (CTL-iPSC-1) or ADA-deficient (ADA-iPSC-1) without or with PEG-ADA was assessed at the indicated days after initiation of early hematopoietic differentiation. **(B)** The number of MHPs generated from CTL-iPSC-2 and ADA-iPSC-2 ± PEG-ADA in day 14 of differentiation. **(C)** The viability of CTL-iPSC-1 and ADA-iPSC-1 as well as CTL-iPSC-2 and ADA-iPSC-2 after 14 days of differentiation, determined by exclusion of PI. Data shown as mean + SD of n = 3 replicates.

Early hematopoietic differentiation: To understand the potential effects of ADA deficiency on early hematopoiesis, 6×10^6^ ADA-deficient and ADA-proficient iPSCs were differentiated into EB. Five days after initiating of differentiation, reductions in the average number of CD34^+^/CD45^+^ MHPs generated from the 6×10^6^ ADA-iPSC-1 were evident in comparison to CTL-iPSC-1 (**Figure 1A**), differences that became more pronounced over time and statistically significant by 11 (p=0.037) and 14 days (p=0.006), respectively. To establish that the reduced number of MHPs from ADA-iPSC-1 was not due to an unappreciated defect in the iPSCs used, MHP formation from ADA-iPSC-2 that were independently created from another patient with identical ADA gene defects was also tested. Similarly significant (p=0.003) reductions in the number of MHPs were also observed in the ADA-iPSC-2 in comparison to CTL-iPSC-2 (**Figure 1B**). Interestingly, the reduction in ADA-deficient cell numbers was not associated with reduced viability of the cells, as determined by PI exclusion (**Figure 1C**, with representative images provided in **Supplementary Figure 3**). PEG-ADA can restore intracellular purine homeostasis by alleviating the ADA substrate accumulation caused by ADA deficiency. Therefore, to establish that the differences observed were directly related to the abnormal Ado metabolism rather than defects in the iPSCs themselves, PEG-ADA was added to the cultures. PEG-ADA treatment significantly increased, and in some instances even normalized, the number of cells generated from ADA-iPSC-1 (p=0.028) and ADA-iPSC-2 cells (p=0.015) in comparison to CTL-iPSC-1 and CTL-iPSC-2, respectively (**Figures 1A, B**).

**Figure 2:**
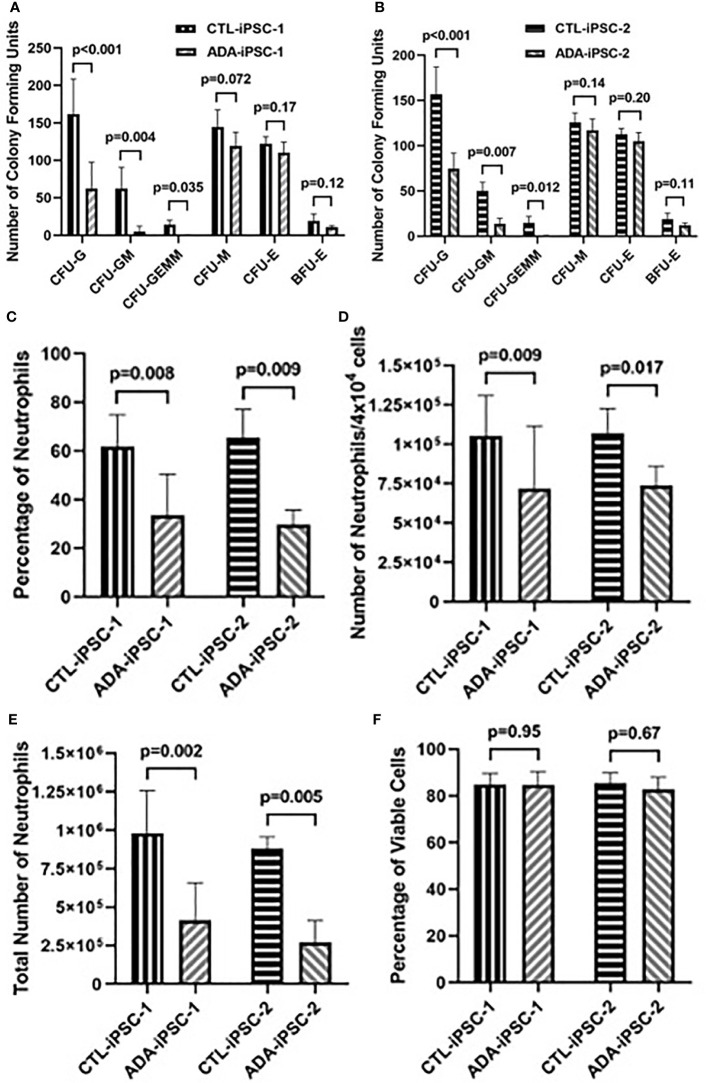
Impaired production of granulocyte colony-forming units and neutrophils from ADA-deficient induced pluripotent stem cells. iPSCs were differentiated for 14 days to form embryoid bodies (EB). The EBs were then dissociated and differentiated in methylcellulose for an additional 12 days to generate hematopoietic colony-forming units (CFU) and neutrophils. The number of CFU of granulocyte (CFU-G), granulocyte-monocyte (CFU-GM), granulocyte-erythroid-monocyte/macrophage (CFU-GEMM), monocyte CFU (CFU-M), and erythroid (CFU-E), as well as erythroid blast-forming units (BFU-E) visualized following 12-day culture in methylcellulose from healthy control (CTL-iPSC-1) and an ADA-deficient patient (ADA-iPSC-1) **(A)**, as well as another control (CTL-iPSC-2) and ADA-deficient patients (ADA-iPSC-2) **(B)**. The percentage of CD11b^+^/CD45^+^ neutrophils **(C)**, the number of neutrophils obtained per 4×10^4^ cells plated in methylcellulose **(D)**, and the total number of neutrophils harvested from methylcellulose **(E)** following the differentiation of 6×10^6^ healthy control (CTL-iPSC-1 and CTL-iPSC-2) or ADA-deficient (ADA-iPSC-1 and ADA-iPSC-2). The percentages of viable (PI^−^) neutrophils generated from ADA-iPSC-1 or CTL-iPSC-1 **(F)**. Data shown as mean + SD of n = 9 replicates for CFU experiments, n = 6 replicates for ADA-iPSC-1- and CTL-iPSC-1-derived neutrophils, and n = 3 for CTL-iPSC-2- and ADA-iPSC-2-derived neutrophils.

Late hematopoietic differentiation and neutrophil characterization: To identify if, in addition to the effects of ADA deficiency on early differentiation, there were also impaired late hematopoiesis and neutrophil development, equal numbers of cells obtained from dissociated 14-day differentiated ADA-deficient and -proficient EBs were cultured in methylcellulose (4×10^4^ cells/well). After additional 12 days in methylcellulose, further exacerbation in the reduction in all granulocyte-containing CFU generated from ADA-iPSC-1 was observed (**Figure 2A**). This was particularly evident in the CFU-G (p<0.001), with an average of 62.7 ± 35.7 colonies/4×10^4^ cells generated from ADA-iPSC-1 compared to 161.7 ± 46.7 colonies/4×10^4^ cells from CTL-iPSC-1. The average numbers of CFU-GM and CFU-GEMM generated from ADA-iPSC-1 were also significantly reduced (p=0.004 and p=0.035, respectively) when compared to CTL-iPSC-1. In contrast, formation of the CFU-E, BFU-E, and CFU-M was not significantly different from CTL-iPSC-1, further confirming that the number of progenitor cells used to initiate these cultures was indeed similar. Similar statistically significant reductions were observed in granulocyte-containing CFU generated from ADA-iPSC-2 in comparison to CTR-iPSC-2 (**Figure 2B**). The percentage of mature neutrophils developed from methylcellulose was determined by analysis of the expression of CD11b and CD45 as well as CD16 and CD66b, which characterize mature neutrophils (31). A significant reduction in the percentage of neutrophils obtained from ADA-iPSC-1 (p=0.008) and ADA-iPSC-2 (p=0.009) in comparison to CTR-iPSC-1 and CTR-iPSC-2, respectively, was evident (**Figure 2C**, with representative flow cytometry plots provided in **Supplementary Figure 4**). This was also reflected in a reduction of the number of neutrophils obtained from ADA-iPSC-1 (p=0.010) and ADA-iPSC-2 (p=0.017) from 4×10^4^ cells/well of methylcellulose (**Figure 2D**). The combined effects of ADA deficiency on early and late development led to a significant reduction in the total number of ADA-deficient neutrophils generated from equal initial numbers (6×10^6^ iPSCs) of ADA-deficient and ADA-proficient iPSCs (**Figure 2E**), with 4.32×10^5^ ± 2.38×10^5^ neutrophils from the ADA-iPSC-1 and 1.04×10^6^ ± 2.54×10^5^ neutrophils from CTL-iPSC-1 (p=0.002). Similar differences were also observed when differentiating ADA-iPSC-2 and CTL-iPSC-2 (p=0.005). Neutrophils isolated from ADA-deficient colonies grown in methylcellulose were frequently indistinguishable from those isolated from ADA-proficient colonies or from the peripheral blood of healthy donors (representative images are provided in **Supplementary Figure 5**). However, the percentage of neutrophils with atypical morphology such as hyperlobulated nucleus was significantly increased (n=4, p=0.003) in cells derived from ADA-iPSC-1 (12.7 ± 0.58%) in comparison to CTL-iPSC-1 (6.66 ± 2.49%). To better understand the cause for reduced numbers of neutrophils generated from ADA-deficient precursors, the viability and apoptosis levels of these cells were assessed. The percentages of PI^−^ live cells (**Figure 2F**) and Annexin V^+^/PI^−^ apoptotic cells (representative images provided in **Supplementary Figure 6**) among ADA-iPSC-1- and CTL-iPSC-1-derived neutrophils were not significantly different.

**Figure 3:**
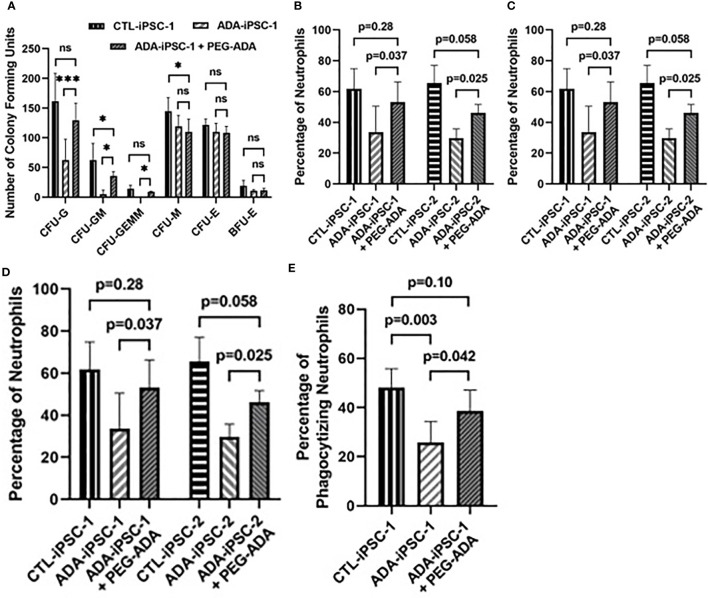
Improved formation of granulocyte colonies and generation of neutrophils from ADA-deficient induced pluripotent stem cells following supplement with ADA. iPSCs were differentiated for 14 days to form EBs. The EBs were then dissociated and differentiated in methylcellulose for an additional 12 days to generate neutrophils, with ADA-deficient iPSCs treated with PEG-ADA replacement enzyme throughout the entirety of the differentiation. The number of Colony-Forming Units (CFU) of granulocyte (CFU-G), granulocyte-monocyte (CFU-GM), granulocyte-erythroid-monocyte/macrophage (CFU-GEMM), monocyte CFU (CFU-M), and erythroid (CFU-E), as well as erythroid blast-forming units (BFU-E) differentiated per 4×10^4^ cells plated in methylcellulose from healthy control (CTL-iPSC-1 and CTL-iPSC-2) or ADA-deficient (ADA-iPSC-1 or ADA-iPSC-2) **(A)**, the percentage of CD11b^+^/CD45^+^ neutrophils **(B)**, and the number of neutrophils obtained per 4×10^4^ cells plated in methylcellulose **(C)**, as well as the total number of neutrophils obtained from an equal initial seeding 6×10^6^ iPSCs **(D)**, and the percentage of neutrophils that have phagocytized red fluorescent microspheres **(E)**, without or with the supplementation of the media and methylcellulose with PEG-ADA. Data shown as mean + SD of **(A)** n = 9 replicates for CFU experiments, n = 6 replicates for CTL-iPSC-1 and ADA-iPSC-1 experiments, and n = 3 for CTL-iPSC-2 and ADA-iPSC-2 experiments; *p < 0.05; ***p < 0.001; ns, not significant.

Restoration of ADA activity: PEG-ADA has been shown to correct the lymphoid immune abnormalities associated with ADA deficiency (38). Therefore, PEG-ADA was added to the methylcellulose cultures to determine the effects of restored purine homeostasis on the generation of neutrophils. Following PEG-ADA treatment, the number of CFU-G colonies generated from ADA-iPSC-1 was significantly (p<0.001) increased (**Figure 3A**). Additionally, the percentage of CD11b^+^/CD45^+^ neutrophils (p<0.05), the number of neutrophils generated per 4×10^4^ cells plated in methylcellulose (p<0.05), and the total number of neutrophils generated from equal initial 6×10^6^ PEG-ADA-treated ADA-iPSC-1 (p=0.018) and ADA-iPSC-2 (p=0.022) were all found to be significantly increased compared to untreated ADA-deficient cells, albeit not to normal levels (**Figures 3B–D**). PEG-ADA treatment (n=4) also significantly decreased (p=0.006) the percentage of ADA-iPSC-1-derived hyperlobular neutrophils from 12.7 ± 0.58% to 9.04 ± 1.68%. Importantly, PEG-ADA also improved the phagocytic function of the neutrophils. Following activation, a significantly reduced (n=5, p=0.003) percentage of ADA-iPSC-1 neutrophils (25.8 ± 8.45%) internalized fluorescent beads in comparison to the healthy CTL-iPSC-1-derived neutrophils (48.0 ± 7.85%). Following PEG-ADA treatment, there was a significant increase (n=5, p=0.042) in the percentage of ADA-iPSC-1 neutrophils capable of phagocytosis (**Figure 3E**).

**Figure 4:**
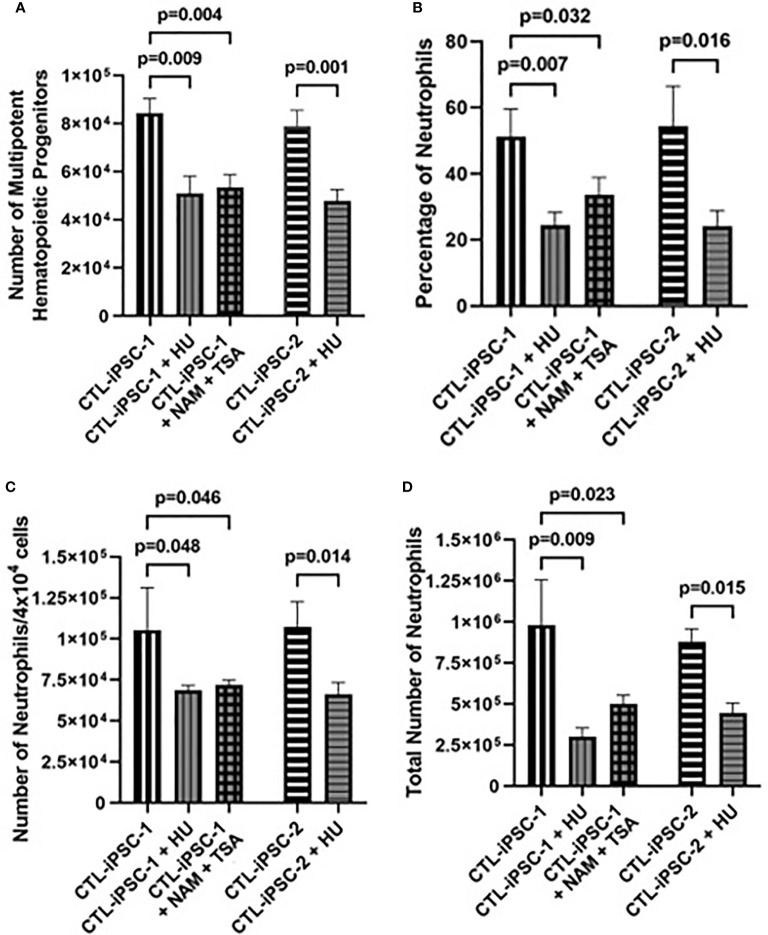
Impaired neutrophil production from induced pluripotent stem cells following inhibition of ribonucleoside reductase. iPSCs were differentiated for 14 days to form EBs. The EBs were then dissociated and differentiated in methylcellulose for an additional 12 days to generate neutrophils, with CTL iPSCs treated with RNR inhibitors HU or NAM + TSA throughout the entirety of the differentiation. The effects of treatment of 6×10^6^ healthy control (CTL-iPSC-1 and CTL-iPSC-2) induced pluripotent stem cells (iPSC) with ribonucleoside reductase inhibitors hydroxyurea (HU) or nicotinamide (NAM) and trichostatin A (TSA) on **(A)** the number of CD34^+^/CD45^+^ multipotent hematopoietic progenitors (MHPs), **(B)** the percentage of CD11b^+^/CD45^+^ neutrophils, **(C)** the number of neutrophils obtained per 4×10^4^ cells plated in methylcellulose, and **(D)** the total number of neutrophils obtained from the 6×10^6^ seeded iPSCs. Data shown as mean + SD of n = 3 replicates.

Role of RNR inhibition in neutrophil development: RNR inhibition secondary to the accumulation of Ado derivatives has been suggested as the cause for the abnormal lymphoid development in ADA deficiency as well as to induce neutropenia among patients receiving various RNR inhibitors. Therefore, the role of RNR was assessed in MHPs and neutrophils generated from ADA-proficient iPSCs (CTL-iPSC-1 and CTL-iPSC-2) treated with the classical RNR inhibitor HU. Following treatment with HU, the total number of MHPs generated from 6×10^6^ CTL-iPSC-1 and CTL-iPSC-2 was significantly decreased (p=0.0092 and p=0.014, respectively) in comparison to the untreated cells (**Figure 4A**), reductions that were similar to those observed in the ADA-deficient iPSCs. Additionally, significant reductions in the percentage of CD11b^+^/CD45^+^ neutrophils (p<0.05), the number of neutrophils generated per 4×10^4^ cells plated in methylcellulose (p<0.05), and the total number of neutrophils generated from the initial 6×10^6^ iPSCs seeded were evident, relative to the untreated CTL-iPSC-1 (p=0.009) and CTL-iPSC-2 (p=0.015) (**Figures 4B–D**). Inhibition of RNR in CTL-iPSC-1 using an alternative combination of the NAM and TSA also significantly reduced (p=0.039) the number of MHPs when compared to the untreated CTL-iPSC-1 (**Figure 4A**). Similarly, significantly reduced percentage of CD11b^+^/CD45^+^ neutrophils (p=0.032), the number of neutrophils generated from 4×10^4^ cells/well of methylcellulose (p=0.046), and the total number of neutrophils generated (p=0.023) were also found following RNR inhibition in CTL-iPSC-1 with NAM and TSA in comparison to untreated cells. The role of RNR for neutrophil development is also suggested by the similar effects on the number of MHPs and the number and percentage of neutrophils generated of ADA deficiency (**Figure 2**) and RNR inhibition (**Figure 4**).

## Discussion

ADA has an important role in the development and function of diverse cells and tissues, and its effects on thymocytes, T cells, chondrocytes, alveolar macrophages, and other cells have been previously studied using patient-derived samples and relevant animal models (7, 43, 44). Additionally, we and others observed neutrophil defects among ADA-deficient patients (14, 15). However, studying the neutrophil abnormalities associated with ADA deficiency have been impeded by the limited access to bone marrow samples as well as peripheral blood samples from affected patients prior to treatment. Additionally, due to substantial differences between human and mouse hematopoiesis ADA-deficient mice do not display aberrations in peripheral blood neutrophil counts (43). Hence, it is not known whether the neutropenia in ADA-deficient patients is due to an intrinsic critical role of ADA in neutrophil development or is secondary to a defect in the bone marrow stroma (18), the frequent infections and immune dysregulation that patients experience or possibly due to treatments they receive (e.g., trimethoprim-sulfamethoxazole). Importantly, deciphering the mechanisms responsible for the neutrophil dysfunction would help understand the role of ADA in myeloid development as well as guide management of ADA-deficient patients. As an alternative research approach, recently developed tools to simulate definitive hematopoiesis *in vitro* and generate myeloid cells and neutrophils from iPSCs were utilized. Furthermore, to ensure that the results observed were not due to additional unknown genetic abnormalities in the patient or unintentional effects of the de-differentiation process, iPSCs established by two different methods (integrated and non-integrated viral vectors) from unrelated patients carrying the same ADA gene defect and healthy controls were used.

Normal ADA function is required for the earliest stages of thymocyte differentiation (45). Similarly, here ADA deficiency was shown to interfere with early hematopoietic development, including the generation of CD34^+^/CD45^+^ MHPs. These results are reminiscent of those recently reported in zebrafish embryos and mouse embryos where Ado signaling has been shown to regulate the first steps of hematopoietic stem and progenitor cell formation (46). Notably, the use of a feeder-free differentiation system avoided potential “detoxification” by ADA-proficient cells of abnormal accumulation of ADA metabolites (47), although it is possible that minimal amounts of residual ADA in the media (48) and/or the frequent media replacements moderated some of the differentiation defects.

In addition to the impaired early hematopoiesis, ADA-deficient cells exhibited significant abnormalities in later development when generated from EBs, apparent by the marked reductions in the generation of CFU-G and CD11b^+^/CD45^+^-expressing neutrophils. Previously, ADA deficiency was shown to cause increased apoptosis of mouse thymocytes (49) and alveolar macrophages (44). In contrast, neutrophils generated from ADA-deficient iPSCs did not exhibit increased apoptosis. This is in line with previous studies that have shown that Ado analogues delay apoptosis of resting human neutrophils in culture, possibly through activation of the A2A AdoR (50). The normal rate of apoptosis also suggests that the reduced number of neutrophils might be due to reduced proliferation or enhanced senescence, as shown previously in ADA-deficient T cells (7, 51). This hypothesis is further supported by the finding here of hypersegmented neutrophils, which can represent enhanced senescence (52).

Interestingly, formation of other erythroid and macrophages/monocytes colonies from ADA-deficient iPSCs were not different than controls, indicating that ADA function was not critical for the development of these cells at this stage. Indeed, anemia is not a typical feature of ADA deficiency, unless induced by autoimmunity (1), and monocyte numbers were previously reported as normal in most ADA-deficient patients (9). The relative resistance of erythroid and macrophages/monocytes development to the effects of abnormal adenosine metabolism might also be due to their expression of different AdoR than neutrophils (53, 54).

In addition to the development and morphological abnormalities, neutrophils generated from ADA-deficient iPSCs have significantly reduced ability to phagocytize fluorescent beads, relative to neutrophils from ADA-proficient iPSCs and from peripheral blood of healthy controls. Indeed, it has previously been postulated that elevated concentrations of Ado inhibit phagocytosis *via* activation of A2A receptors (51). The impaired development and function of iPSC-derived ADA-deficient neutrophils was only partial, possibly reflecting either the relatively limited accumulation of toxic purine metabolites in the *ex vivo* system or the relatively short duration of exposure to these metabolites or additional unknown mechanisms. Yet, the findings here are similar to those observed in ADA-deficient patients who often display mild-moderate neutrophil dysfunction that might progress over a period of several weeks, unless exacerbated by infections or medications (14).

Exogenous supplementation with PEG-ADA has been shown to reverse many of the *in vitro* and *in vivo* abnormalities caused by ADA deficiency. Similarly, the addition of PEG-ADA to the differentiation media and methylcellulose normalized the formation of granulocyte colonies and neutrophils, as well as rescued the function of these neutrophils. These findings further demonstrate that indeed the abnormalities observed in the MHPs and neutrophils generated from the ADA-deficient patient-derived iPSCs were directly caused by the inherited metabolic defect. Moreover, although the concentration of Ado and dAdo in the various compartments was not determined, the ability of PEG-ADA to correct the neutrophil morphological and functional abnormalities implicates the impaired purine homeostasis as the cause of the abnormalities. This is because PEG-ADA can’t cross the cellular membrane and acts by lowering Ado and dAdo outside of the cells and subsequently inside the cells, following the exit of purines in accordance to their concentration gradient through nucleoside transporters. The ability of PEG-ADA supplement to improve the generation of neutrophils *in vitro* corroborates the clinical practice of treating acutely ill ADA-deficient SCID patients with enzyme replacement. Additionally, the ability of PEG-ADA to increase the number of CD34^+^ MHPs further supports the administration of PEG-ADA prior to harvesting bone marrow cells from ADA-deficient that are used for autologous gene therapy (17).

Accumulation of dATP in ADA deficiency has previously been shown to inhibit the activity of RNR in T lymphocytes (55). To identify whether a similar mechanism contributed to the neutrophil abnormalities in ADA deficiency, RNR was inhibited in ADA-proficient iPSC. RNR is indispensable for cell survival; therefore, RNR function was suppressed while preserving the viability of the cells. Indeed, the effects of RNR inhibition including reduction in the production of MHPs and neutrophils were similar to those observed in ADA-deficient iPSCs. Interestingly, treating CD34^+^ cells with a combination of NAM and NAM phosphoribosyltransferase (NAMPT) was previously shown to enhance myelopoiesis, possibly by upregulating the expression G-CSF and G-CSF receptor (56). However, others have also shown that high concentrations of NAM without supplementing NAMPT promote myelosuppression (57, 58) and RNR inhibition (21), as demonstrated here.

This study has several limitations. Although iPSC-derived neutrophils share many characteristics of mature neutrophils, as demonstrated here, there have been reports of reduced phagocytosis, decreased formation of neutrophil extracellular traps, and altered signaling, indicating that the *ex vivo* generated cells might not fully recapitulate the behavior of primary cells (31). Moreover, while the current study evaluated iPSC-derived cells from two ADA-deficient infants, the number of patients limits the broader interpretation of the results as it is possible that patients with other ADA gene mutations will exhibit a different hematopoietic phenotype. Accordingly, examining iPSCs from additional patients, and possibly correlating the results with those obtained from peripheral blood neutrophils, will be beneficial. In the future, direct demonstration of defective RNR activity in patient’s neutrophils and correction of HU-mediated inhibition in ADA-deficient MPH and neutrophil with PEG-ADA supplementation will help support the hypothesis that RNR dysfunction is the cause for neutrophil abnormalities in ADA deficiency.

In conclusion, our study provides further evidence that ADA is required for hematopoietic and neutrophil development and that the neutropenia exhibited by ADA-deficient patients is directly associated with the enzyme defect. Hence, neutrophil abnormalities should be added to the expanding list of effects caused by abnormal ADA activity.

## Data Availability Statement

The original contributions presented in the study are included in the article/**Supplementary Material**. Further inquiries can be directed to the corresponding author.

## Ethics Statement

The studies involving human participants were reviewed and approved by Research Ethics Board of the Hospital for Sick Children, Toronto, Ontario and by the National Institutes of Health Institutional Review Board (protocol 16-I-N139). Written informed consent to participate in this study was provided by the participants’ legal guardian/next of kin.

## Author Contributions

EG, YD, and MT conceptualized this study and designed the experiments. All authors contributed to writing, reviewing the manuscript, and data visualization. MT and WM conducted the experiments. All authors contributed to the article and approved the submitted version.

## Funding

This work was supported in part by the Campbell Chair for Immunology Research (EG) and by the SickKids Food Allergy and Anaphylaxis Program (EG) and by the Division of Intramural Research, National Institute of Allergy and Infectious Diseases (grant ZIA AI001222 to LN).

## Conflict of Interest

The authors declare that the research was conducted in the absence of any commercial or financial relationships that could be construed as a potential conflict of interest.

## Publisher’s Note

All claims expressed in this article are solely those of the authors and do not necessarily represent those of their affiliated organizations, or those of the publisher, the editors and the reviewers. Any product that may be evaluated in this article, or claim that may be made by its manufacturer, is not guaranteed or endorsed by the publisher.

## Supplementary Material

Supplementary Figure 1 Expression of characteristic cell surface markers by neutrophils generated from induced pluripotent stem cells. Representative FACS histogram plots showing expression of CD11b, CD45, CD16, and CD66b by neutrophils isolated from peripheral blood of healthy donors (lower panel) or neutrophils generated from iPSCs and subsequently isolated from methylcellulose (upper panel). Characterization of the CD11b+/CD45+ population demonstrates that these cells maintain greater than 95% CD16/CD66b expression, identifying them as neutrophils.

Supplementary Figure 2 Reactive oxygen species formation by neutrophils generated from induced pluripotent stem cells. Representative FACS histogram plots showing fluorescence resulting from the oxidation of cell permeant reagent dihydrorodhamine 123 in neutrophils generated from iPSCs and subsequently isolated from methylcellulose (left panel) or healthy donor peripheral blood neutrophils (middle panel) as well as healthy donors’ peripheral blood CD4+ T cells (right panel) prior to and after stimulation with PMA.

Supplementary Figure 3 Viability of multipotent hematopoietic progenitors. Representative FACS histogram plots showing PI staining in multipotent hematopoietic progenitors generated from CTL-iPSC-1, ADA-iPSC-1, CTL-iPSC-2, and ADA-iPSC-2.

Supplementary Figure 4 Reduced percentage of neutrophils generated from ADA-deficient induced pluripotent stem cells. Representative flow cytometry graphs depicting the percentages of CD11b^+^/CD45^+^ neutrophils obtained from methylcellulose following differentiation from CTL-iPSC-1 and ADA-iPSC-1 as well as ADA-iPSC-1 treated with PEG-ADA. Additional figures depict the percentage of CD11b and CD45 expression in neutrophils and T-cells of the peripheral blood from a healthy control.

Supplementary Figure 5 Images of neutrophils generated from ADA-deficient and healthy control induced pluripotent stem cells. Representative images of May-Giemsa-Grunewald-staining of neutrophils derived from iPSCs of a healthy control subject (upper figures) and an ADA-deficient patient (lower figures). Neutrophils derived from ADA-deficient iPSCs demonstrate prominent hyperlobulation (≥6 lobes).

Supplementary Figure 6 Apoptosis of neutrophils generated from ADA-deficient and healthy control induced pluripotent stem cells. Representative FACS histogram plots showing annexin V/PI staining in neutrophils generated from ADA-iPSC-1 or CTL-iPSC-1.

## Abbreviations

ADA, adenosine deaminase; ADA-iPSC, induced pluripotent stem cell lines derived from ADA-deficient patients; Ado, adenosine; AdoR, adenosine receptors; BFU-E, erythrocyte blast-forming unit; CFU, colony-forming unit; CFU-E, erythrocyte colony-forming unit; CFU-G, granulocyte colony-forming unit; CFU-GEMM, granulocyte erythrocyte monocyte megakaryocyte colony-forming unit; CFU-GM, granulocyte monocyte colony-forming unit; CFU-M, monocyte colony-forming unit; dAdo, deoxyadenosine; dATP, deoxyadenosine triphosphate; EB, embryoid body; HSC, hematopoietic stem cell; HU, hydroxyurea; iPSC, induced pluripotent stem cell; MHP, multipotent hematopoietic progenitor; NAM, nicotinamide; NAMPT, nicotinamide phosphoribosyltransferase; PI, propidium iodide; RNR, ribonucleotide reductase; SCID, severe combined immunodeficiency; TSA, trichostatin A.
