# Supplementary figures and images for: The Use of Induced Pluripotent Stem Cells to Study the Effects of Adenosine Deaminase Deficiency on Human Neutrophil Development
Source: Front Immunol. 2021 Oct 28;12:748519. doi: 10.3389/fimmu.2021.748519 (PMC8582638; doi:10.3389/fimmu.2021.748519)

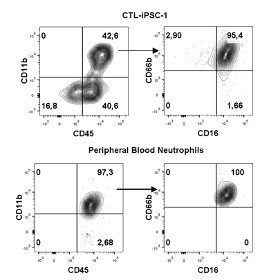

Supplement: Supplementary Figure 1 — Expression of characteristic cell surface markers by neutrophils generated from induced pluripotent stem cells. Representative FACS histogram plots showing expression of CD11b, CD45, CD16, and CD66b by neutrophils isolated from peripheral blood of healthy donors (lower panel) or neutrophils generated from iPSCs and subsequently isolated from methylcellulose (upper panel). Characterization of the CD11b+/CD45+ population demonstrates that these cells maintain greater than 95% CD16/CD66b expression, identifying them as neutrophils. [file Image_1.jpeg]

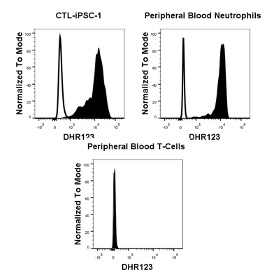

Supplement: Supplementary Figure 2 — Reactive oxygen species formation by neutrophils generated from induced pluripotent stem cells. Representative FACS histogram plots showing fluorescence resulting from the oxidation of cell permeant reagent dihydrorodhamine 123 in neutrophils generated from iPSCs and subsequently isolated from methylcellulose (left panel) or healthy donor peripheral blood neutrophils (middle panel) as well as healthy donors’ peripheral blood CD4+ T cells (right panel) prior to and after stimulation with PMA. [file Image_2.jpeg]

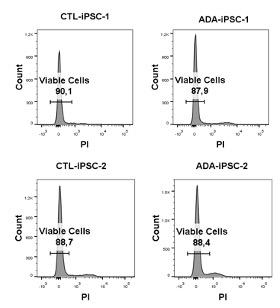

Supplement: Supplementary Figure 3 — Viability of multipotent hematopoietic progenitors. Representative FACS histogram plots showing PI staining in multipotent hematopoietic progenitors generated from CTL-iPSC-1, ADA-iPSC-1, CTL-iPSC-2, and ADA-iPSC-2. [file Image_3.jpeg]

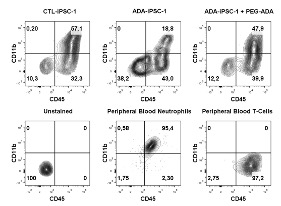

Supplement: Supplementary Figure 4 — Reduced percentage of neutrophils generated from ADA-deficient induced pluripotent stem cells. Representative flow cytometry graphs depicting the percentages of CD11b+/CD45+ neutrophils obtained from methylcellulose following differentiation from CTL-iPSC-1 and ADA-iPSC-1 as well as ADA-iPSC-1 treated with PEG-ADA. Additional figures depict the percentage of CD11b and CD45 expression in neutrophils and T-cells of the peripheral blood from a healthy control. [file Image_4.jpeg]

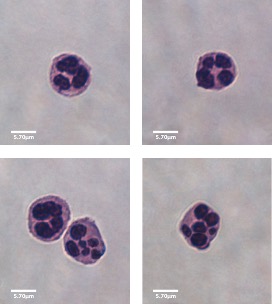

Supplement: Supplementary Figure 5 — Images of neutrophils generated from ADA-deficient and healthy control induced pluripotent stem cells. Representative images of May-Giemsa-Grunewald-staining of neutrophils derived from iPSCs of a healthy control subject (upper figures) and an ADA-deficient patient (lower figures). Neutrophils derived from ADA-deficient iPSCs demonstrate prominent hyperlobulation (≥6 lobes). [file Image_5.jpeg]

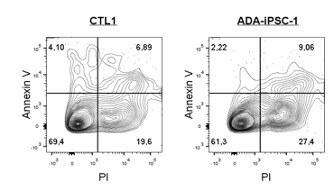

Supplement: Supplementary Figure 6 — Apoptosis of neutrophils generated from ADA-deficient and healthy control induced pluripotent stem cells. Representative FACS histogram plots showing annexin V/PI staining in neutrophils generated from ADA-iPSC-1 or CTL-iPSC-1. [file Image_6.jpeg]
